# Luxation of Humerus from Muscular Contraction

**Published:** 1872-08-15

**Authors:** A. H. Salisbury

**Affiliations:** Mazomanie, Wis.


### LUXATION OF HUMERUS FROM MUSCULAR CONTRACTION.

BY A. H. SALISBURY, M.D., MAZOMANIE, WIS.

*Editors Medical Examiner:*—Allow me to report a case coming under my observation, not so much for its value, but because it is somewhat unique.

Mr. G. T——, a strong, muscular man, well formed; weight over two hundred pounds; age about forty; complexion dark; has for the last two years been subject to epileptic convulsions, at intervals of two or three months. In December last he had an attack after which he complained of a lameness in the right shoulder, but which disappeared in a few days.

April 19. I was called in the night. When I reached his house, the paroxysm was over, and I found him sitting up in bed nursing his right arm, and complaining of pain in the right shoulder. He had gone to bed about twelve o'clock, and after lying on his back for a few minutes, turned upon his left side, when the attack immediately came on, the muscles of the right side contracting strongly. The pain not subsiding, I administered morphine and chloral hydrate, and after a short time, injected morphine subcutaneously.

This quieted the pain and he went to sleep and slept most of the next day and night. I made no examination, for I supposed the pain arose from the same cause as in the previous attack—to violent muscular contraction.

April 21. When I called he was up and dressed, but said the pain in the shoulder still troubled him. I suggested an examination, but as he did not seem disposed, I ordered some anodyne liniment and left him.

On the 22d, being no better, I insisted upon an examination, and upon uncovering the shoulder, found a backward dislocation of humerus, the head resting on the dorsum scapulæ. By using slight extension and a little manipulation, the head slipped into place with a snap, giving my patient instant relief. He has had one attack since, but no trouble of this kind.

